# SPRR2A enhances p53 deacetylation through HDAC1 and down regulates p21 promoter activity

**DOI:** 10.1186/1471-2199-13-20

**Published:** 2012-06-25

**Authors:** Yoshiaki Mizuguchi, Susan Specht, John G Lunz, Kumiko Isse, Natasha Corbitt, Toshihiro Takizawa, Anthony J Demetris

**Affiliations:** 1 Thomas E. Starzl Transplantation Institute, University of Pittsburgh Medical Center, Pittsburgh, PA, 15260, USA; 2 The Department of Pathology, University of Pittsburgh Medical Center, Pittsburgh, PA, 15260, USA; 3 Surgery, Divisions of Transplantation, University of Pittsburgh Medical Center, Pittsburgh, PA, 15260, USA; 4 Department of Molecular Anatomy and Medicine, Nippon Medical School, 1-1-Sendagi, Bunkyo-Ku, Tokyo, 113-8602, Japan

## Abstract

**Background:**

Small proline rich protein (SPRR) 2A is one of 14 SPRR genes that encodes for a skin cross-linking protein, which confers structural integrity to the cornified keratinocyte cell envelope. New evidence, however, shows that SPRR2A is also a critical stress and wound repair modulator: it enables a variety of barrier epithelia to transiently acquire mesenchymal characteristics (EMT) and simultaneously quench reactive oxygen species during wound repair responses. p53 is also widely recognized as the node in cellular stress responses that inhibits EMT and triggers cell-cycle arrest, apoptosis, and cellular senescence. Since some p53-directed processes would seem to impede wound repair of barrier epithelia, we hypothesized that SPRR2A up regulation might counteract these effects and enable/promote wound repair under stressful environmental conditions.

**Results:**

Using a well characterized cholangiocarcinoma cell line we show that levels of SPRR2A expression, similar to that seen during stressful biliary wound repair responses, disrupts acetylation and subsequent p53 transcriptional activity. p53 deacetylation is accomplished via two distinct, but possibly related, mechanisms: 1) a reduction of p300 acetylation, thereby interfering with p300-p53 binding and subsequent p300 acetylation of K382 in p53; and 2) an increase in histone deacetylase 1 (HDAC1) mRNA and protein expression. The p300 CH3 domain is essential for both the autoacetylation of p300 and transference of the acetyl group to p53 and HDAC1 is a component of several non-p300 complexes that enhance p53 deacetylation, ubiquitination, and proteosomal degradation. HDAC1 can also bind the p300-CH3 domain, regulating p300 acetylation and interfering with p300 mediated p53 acetylation. The importance of this pathway is illustrated by showing complete restoration of p53 acetylation and partial restoration of p300 acetylation by treating SPRR2A expressing cells with HDAC1 siRNA.

**Conclusion:**

Up-regulation of SPRR2A, similar to that seen during barrier epithelia wound repair responses reduces p53 acetylation by interfering with p300-p53 interactions and by increasing HDAC1 expression. SPRR2A, therefore, functions as a suppressor of p53-dependent transcriptional activity, which otherwise might impede cellular processes needed for epithelial wound repair responses such as EMT.

## Background

p53 is a nodal convergence point of integrated intra-cellular signaling networks that mediate cellular responses to stress (e.g. oxidative stress or DNA damage). It regulates expression of many stress-related target genes and their proteins, such as p21, GADD45, Bax, Puma, and Noxa, by binding to the p53 response element (RE) in their promoter regions [1]. p53 is tightly regulated, however, as a “cellular gatekeeper” [2] and the three-step activation process of p53 is complex: stabilization, DNA binding, and transcriptional activation [3]. As many as 50 individual posttranslational modifications contribute to or influence the ability of p53 to function as a sequence-specific transcription factor during normal homeostasis and stress-induced responses [4,5].

p53 activation is also modulated by transcriptional co-activators (e.g. p300 [6]) and inhibited by a variety of proteins, such as MDM4 and MDM2, which ubiquitinates p53 targeting it for proteasome-mediated degradation. Thus, p53 and MDM2 form a negative feedback regulatory loop. MDM2-mediated p53 destruction is synergistic with histone deacetylase 1 (HDAC1): these molecules often complex together, coupling p53 deacetylation and ubiquitination [7].

p53 is also subject to, and exerts, cytoplasmic influences [8]. p53 phosphorylation by kinases (e.g. ATM/ATR/DNAPK), and Chk1/Chk2 is regarded as the first crucial step in p53 stabilization [3]. Post-translational p53 acetylation helps regulate protein concentrations and transcriptional activity [9]. Cellular stress (e.g. H_2_O_2_) and over expression of p300/CBP [6] causes K382-p53 acetylation and p53 protein accumulation [6]. The latter also results in increased sequence specific p53-DNA binding [10]. Other p53 lysine modifications such as methylation, ubiquitination, sumoylation, and neddylation also have the potential to alter p53’s transcriptional activity [4,5].

Typically, p53 enhanced transcriptional activity increases p21 expression during cellular stress, which in turn, blocks cell cycle progression and inhibits proliferation. p53 activation can also block epithelial-to-mesenchymal transition (EMT) via upregulation of miR-200 and miR-192 family members that repress ZEB1/2 expression [11], which are key mediators of EMT. Paradoxically, these p53-directed stress responses, p21 upregulation and EMT blockage, are at odds with the two main processes needed in the epithelia for wound repair: proliferation and migration [12].

Small proline rich protein (SPRR) 2A, one of 14 SPRR genes coded in the region of the epidermal differentiation complex [13], is coordinately expressed with other genes in the complex. In the normal skin it functions primarily as a keratinocyte cross-linking protein that confers structural integrity to the cornified cell envelope [14]. Exciting new evidence, however, shows non-coordinate, independent up-regulation of SPRR proteins occurs almost universally in a variety of pathophysiological conditions involving stress and wound repair in the barrier epithelia ( [15]). Remaining viable epithelial cells at the edges of wounds transiently undergo epithelial-mesenchymal transition (EMT) [15,16], a process essential for the restitution/migration phase of epithelial wound healing [12].

Previous data from our group showed that forced expression of SPRR2A in the cholangiocarcinoma cell line SG231, at levels similar to those seen during wound repair responses, induced EMT and significantly reduced cell death under H_2_O_2_- and glycochenodeoxycholate-induced cell injury [15]. Parallel observations were made in keratinocytes [17]. Therefore, beyond its role in skin cornification, SPRR proteins have a widespread role in tissue remodeling and function as global links between ROS detoxification and cell migration during wound healing [18]. These observations prompted us to test the hypothesis that stress induced non-coordinate upregulation of SPRR2A in barrier epithelia counteracts the transcriptional activity of p53, thereby enabling cellular adaptations needed for normal wound repair under stressful circumstances.

## Results and discussion

### SPRR2A blocks acetylation of K382-p53

**Figure 1:**
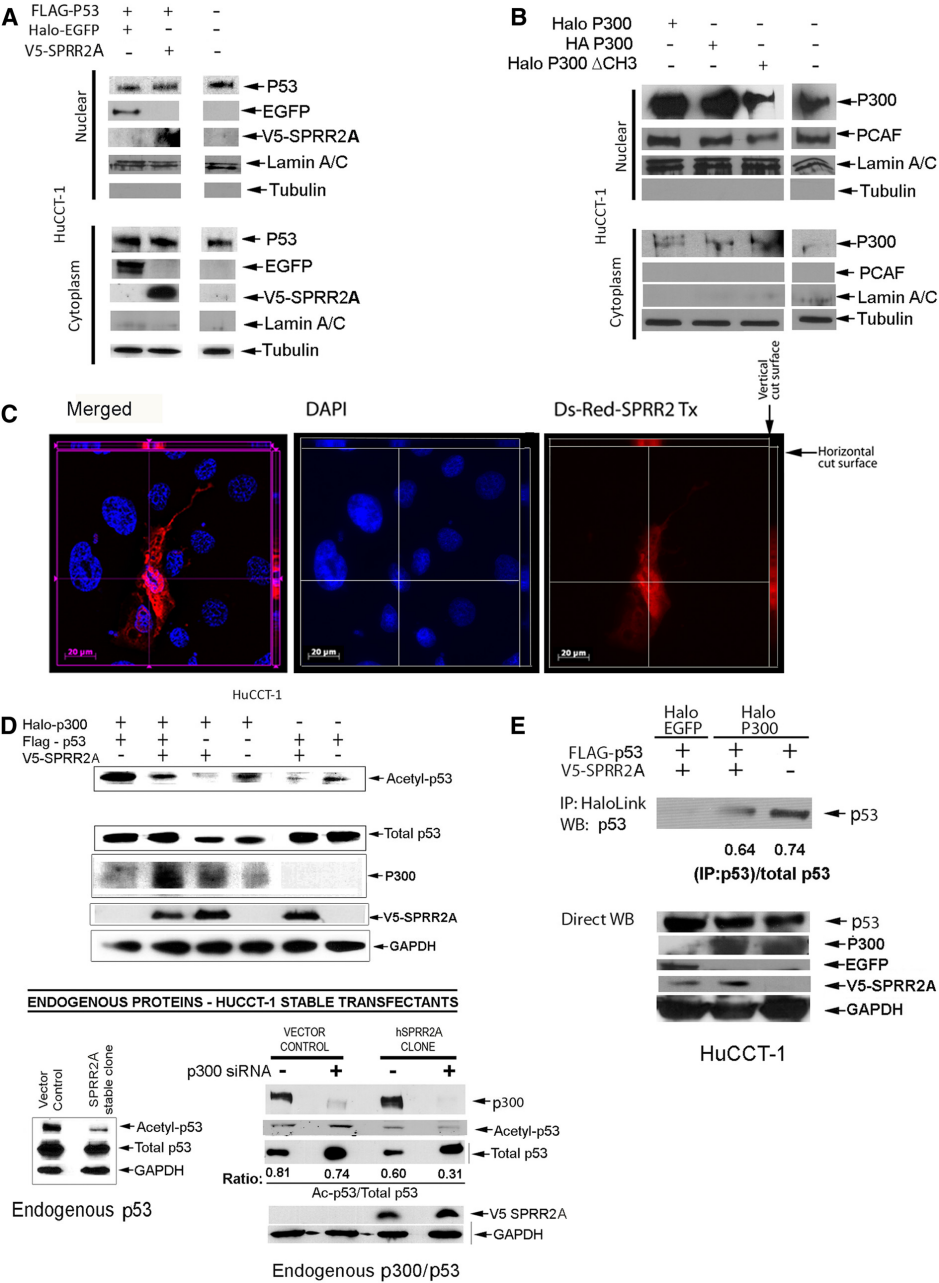
**SPRR2A blocks p300 induced acetylation of K382-p53.** (**A**)Western blotting of nuclear and cytoplasmic protein obtained from HuCCT-1 cells 48 hours post-transfection with V5-SPRR2A, and FLAG-p53 plasmids. SPRR2A and p53 were detected in both the cytoplasm and nucleus. SPRR2A expression did not change the distribution of p53. Halo-EGFP vector was used to adjust vector amounts. Lamin A/C and Tubulin were used to verify nuclear and cytosolic separation, respectively. (**B**) Western blot analysis of the distribution of acetyltransferases p300 and PCAF protein 48 hours after transfection with the indicated vectors. P300 and its cysteine/histidine rich region 3 (ΔCH3) deletion construct distribute primarily to the nucleus, with some protein detected in the cytoplasm. PCAF was only detected in the nucleus. Lamin A/C and tubulin were used to verify separation of nuclear and cytoplasmic fractions. (**C**) HuCCT-1 cells transfected with a DsRed-SPRR2A plasmid and SPRR2A protein visualized via Axiovision, an advanced, automated imaging system (Carl Zeiss, Gottingen, Germany). Axiovision software enables Z-stack capture (10 stack levels/0.35 micron spacing per level). In the vertical cut surface, the cytoplasm is positive for DsRed-SPRR2A expression. The horizontal cut surface shows DsRed-SPRR2A co-localized with DAPI staining in the nucleus. (**D**) Upper panel: Western blotting of Ac-K382-p53 in cells transfected with indicated vectors shows that SPRR2A reduces Ac-K382-p53, both with and without p300 over-expression. Lower panel, left: A SPRR2A stable transfectant also shows deacetylation of p53 without p53 or p300 over-expression. Lower panel, right: EP300 siRNA further reduced Ac-K382-p53 levels in the SPRR2A stable transfectant, suggesting acetylation of p53 occurs through both p300 and non-p300 mediated pathways. (**E**) Immunoprecipitation of p300 shows that the presence of SPRR2A reduces p300/p53 binding.

We first determined whether SPRR2A protein expression in HuCCT-1 cells altered the distribution of Flag-tagged p53 transfected protein, which it did not. p53 and SPRR2A proteins were detected in the nucleus and cytoplasm (Figure 1A**)**, but SPRR2A did not change the distribution of p53. In contrast, p300 and its cysteine/histidine-rich (CH) region 3 deletion construct distributed primarily to the nucleus, but low-level cytoplasmic localization was also seen (Figure 1B). Cytoplasmic p300 can ubiquinate p53 and target it for destruction thereby preventing cytoplasmic p53 accumulation [19]. The intra-cellular distribution of SPRR2A was confirmed by expression of a Ds-Red-SPRR2A construct that showed both nuclear and cytoplasmic protein expression in HuCCT-1 cells (Figure 1C). 

Simultaneous over expression of p53 and p300 significantly increased the level of Ac-K382-p53, indicating that in HuCCT-1 cells, p53 acetylation involves p300 (Figure 1D). Co-transfection of HuCCT-1 with combinations of SPRR2A, p300, and p53 vectors showed the following: 1) In the presence of p300 over expression, SPRR2A caused a decrease in Ac-K382-p53, both with and without p53 transfection. 2) SPRR2A transfection decreased p53 acetylation in the absence of p300 over expression, suggesting that SPRR2A also influences p53 acetylation/stabilization through other non-p300 related mechanisms (Figure 1D).

To verify that the SPRR2A reduction in Ac-K382-p53 was not a consequence of p53 and/or p300 over expression, we used a cell line stably transfected with SPRR2A alone to determine the effects on endogenous p53. The SPRR2A clone showed a marked reduction in endogenous Ac-K382-p53 when compared to its vector control (Figure 1D; lower panel, left). HuCCT-1 cells harbor a point mutation (H175) in p53, which reduces (but does not eliminate) binding to the p21 promoter ( http://p53.free.fr/Database/Cancer_cell_lines/p53_cell_lines.html). Therefore, SPRR2A is capable of decreasing acetylation of both endogenous (mutant) p53 and transfected (wt) p53 (Figure 1D). To verify that the reduced acetylation seen with transfected (wt) p53 was not influenced by the presence of mutant p53 in HuCCT-1 cells, we examined the effect of SPRR2A over-expression in a cell line with (wt) p53. Like HuCCT-1 cells, the human hepatoma cell line HepG2 does not express SPRR2A (Additional file 1: Figure S1A**)** and HepG2 endogenous p53 is wild type [20]**.** Transient transfection of SPRR2A in HepG2 cells resulted in a marked reduction of K-382-p53 acetylation and a corresponding reduction in p21 mRNA **(**Additional file 1: Figure S1B-C**)**, confirming a role for SPRR2A in the acetylation and transactivation of p53.

To determine if the SPRR2A-induced p53 deacetylation was p300 dependent, we knocked down endogenous p300 expression with siRNA (Figure 1D; lower panel, right). In both the vector control and SPRR2A clone, removal of p300 resulted in an increase in total p53, as previously reported [21] and is attributed to the role of p300 in the removal of p53 through ubiquitination and proteasomal targeting [19,22]. In the vector control, loss of p300 causes a slight increase in Ac-K382-p53, but the ratio of Ac-K382-p53/total p53 is maintained through compensatory p300 independent mechanisms (Figure 1D: lower panel right). If SPRR2A interferes with p53 acetylation solely through p300, knocking out p300 should restore Ac-K382-p53 levels to those seen in the siRNA treated vector control. Likewise, if SPRR2A does not interfere with p300 acetylation of p53, p300 knock down should not alter the Ac-K382-p53/total p53 ratio seen in the clone.

Results showed that p300 knock down in the SPRR2A clone yielded a relative reduction in Ac-K382-p53 when compared to the total p53 in the cell. This would occur if SPRR2A reduces not only p300 directed acetylation of p53 (which is further reduced by EP300 siRNA), but also blocks the compensatory p300 independent pathway that maintains Ac-K382-p53/total p53 levels in the vector control (Figure 1D). The same change in Ac-K382-p53 with EP300 siRNA was obtained in two other stable SPRR2A clones (data not shown). p300 acetylation of p53 requires direct interaction between these two proteins and immunoprecipitation experiments showed that SPRR2A expression inhibits p300-p53 binding (Figure 1E).

We also considered that SPRR2A might bind directly to p300 or p53 and interfere with subsequent acetylation, but immunoprecipitation experiments failed to show any direct interaction (data not shown). Consequently, the observed effect is likely upstream of these molecules.

Altogether our observations suggest that SPRR2A prevents acetylation of K382-p53 in two ways: the first involves p300: SPRR2A dissociates or blocks p300-p53 binding, which in turn prevents acetylation of K382-p53 by p300; the second is p300-independent: SPRR2A acts through other p53 regulators to reduce the activation/stabilization of p53. Since deacetylated p53 is less stable and more readily degraded, SPRR2A stable clones have less total p53 (Figure 1D), suggesting that SPRR2A expression yields less Ac-K382-p53 by enhancing ubiquitination and degradation.

### SPRR2A down-regulates p53-DNA binding and target gene transcription

**Figure 2:**
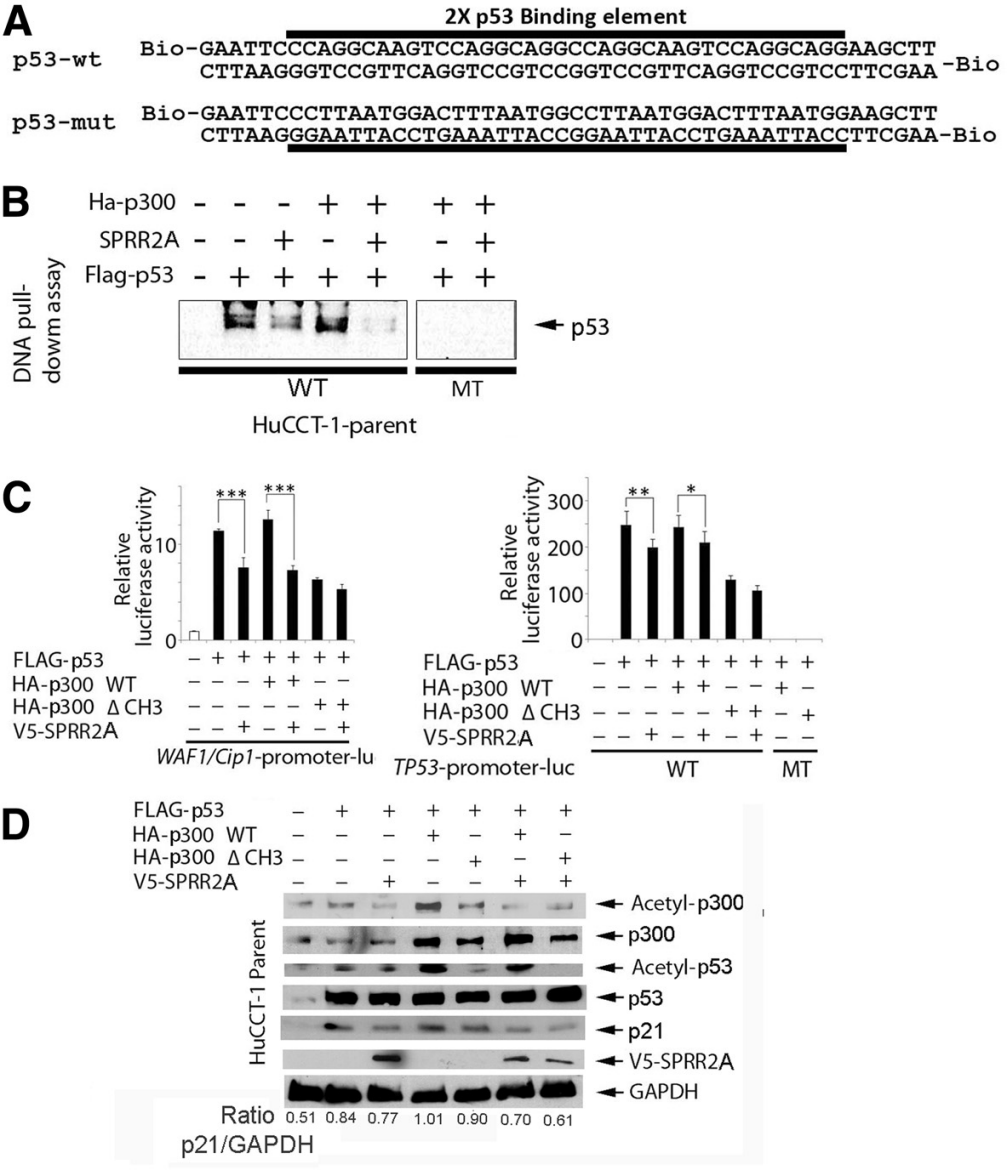
**SPRR2A dislocates p53 from its DNA binding element, reducing target gene transcription.** (**A**) Sequence for the biotinylated double-stranded DNA probes that mimic the wild type and mutational p53 response elements (RE). (**B**) DNA pull-down assay showing that SPRR2A expression reduces p53 binding to the p53-RE when compared to its corresponding control. That is, p53 binding strengths are as follows: transfection with p53 > p53/SPRR2A and transfection with p53/p300 > p53/p300/SPRR2A. (**C**) Luciferase assays of p21-RE and p53-RE from cells transfected with indicated vectors. For p53-RE reporter, we used wild type (WT) and mutational (MT) RE vectors**.** Again, SPRR2A expression reduced reporter activity when compared to the corresponding control transfection. (**D**) Western blot analysis showing that SPRR2A reduced p21 expression and acetylation of p300 and p53. WT, wild type; MT, mutation; *, p < 0.05; ** <0.01, *** < 0.001.

Next, we determined whether SPRR2A expression influences p53 DNA binding activity using biotinylated double-stranded oligonucleotide probes that mimic the wild type or mutational sequences of known p53 binding motifs [23]. Lysates from HuCCT-1 parent cells, transfected with p53 vectors, show that p53 can bind with the intact, wild type p53 response element (RE), but not the mutated RE, indicating that p53 binding is sequence specific (Figure 2A and 2B). Over expression of p300 only slightly increased p53 binding to this element (verified by imageJ analysis), most likely because the binding element is not in the context of the genome where DNA conformation and upstream/downstream co-factor binding influences p53 binding. Co-existent SPRR2A expression in the protein lysate, however, decreased p53 binding to the element when compared to its corresponding control: P53 > p53/SPRR2A; p53/p300 > p53/p300/sprr2A. Furthermore, SPRR2A significantly reduced this p53/RE binding in the absence of p300 over expression, supporting a role for SPRR2A in regulating p53 through non-p300 mechanisms (Figure 2B). DNA pull down assays using wild type p53 RE motif did not show any direct binding of SPRR2A, indicating that SPRR2A does not act as a transcription factor that competes with p53 for binding to the response element (results not shown). These results are in accordance with the above hypotheses suggesting that the effect of SPRR2A on K382-p53 acetylation is what modulates p53 DNA binding. 

These observations, however, still do not determine whether SPRR2A and/or p300-mediated changes in p53 acetylation and DNA-binding affect p53 target gene transcription. p53 regulates p21 gene expression by directly binding to a p53-RE on the p21 promoter region [23], followed by recruitment of p300/CBP and acetylation of p53 [24]. We examined transcriptional activity using a luciferase reporter vector containing the p21 promoter. As shown in Figure 2C, over expression of p53 in HuCCT-1 cells significantly increased the *p21* promoter activity, as expected. In addition, this effect was increased by co-transfection with a wild type p300 vector, but in this reporter system it did not reach statistical significance. SPRR2A expression decreased *p21* promoter activity significantly, with and without p300 over expression, supporting previous data (Figure 1D) showing that SPRR2A affects not only p300, but other p53 regulators as well. Although less effective, a luciferase assay using p53-RE-luc and its mutational construct demonstrated a similar reduction in activity after SPRR2A expression (Figure 2C). These results show that SPRR2A can affect transcription not only on the *p21* promoter, but on other promoters with a p53-RE as well.

To corroborate the above hypothesis suggested by the luc-reporter assays, *in vivo* protein expression profiles were examined following similar transfections in parent HuCCT-1 (Figure 2D). Although the p21-luc reporter did not yield a significant increase in p21 transcription following combined p53/p300 transfection, Figure 2D shows that transfection of both p53 and p300 increases p21 expression *in vivo*. Furthermore, compared to wild type p300, less Ac-K83-p53 and p21 protein is expressed if ΔCH3 p300 is transfected. And finally, all p21 levels are reduced in the presence of SPRR2A.

Insights into how SPRR2A interacts with p300 to inhibit p53 DNA binding are seen in Figure 2D. Wild type-p300 is acetylated in HuCCT-1-parent cells, but SPRR2A induction de-acetylated p300, indicating a possible mechanism of SPRR2A’s suppressive effect on p21 transcription (Figure 2D). p53 protein can bind to both the CH1 and CH3 sites on p300, but the binding sequences for each are different [25]. The CH3 site interacts with many transcription factors, including p53 [26-28]. Similar to SPRR2A induction, transfection with a CH3-deleted p300 vector reduced promoter activity when compared to wild type p300 **(**Figure 2C**)**. And in accordance with the promoter assays, transfection with a CH3-deleted p300 vector also diminished the level of Ac-K382-p53 and p21 (Figure 2D). Since CH3-deleted p300 protein was not acetylated, even in the absence of SPRR2A in HuCCT-1 cells, the CH3 domain appears to be crucial for p300 acetylation followed by p53 acetylation (Figure 2D). Moreover, expression of SPRR2A does not exert an additional suppressive effect on promoter activity in the CH3-deleted p300 expressing cells (Figure 2C-D**)**. This suggests that the effects of SPRR2A (i.e., reduced Ac-K382-p53, Ac-p300 and p53-RE gene transcription) require a functional CH3 domain on p300.

### HDAC1 reduces p53 acetylation in SPRR2A cells

Previous data from our lab showed that SPRR2A functions as a SH3 domain ligand using its xPxxP motifs [15] and the p300 CH3 domain can bind to a xPxxP motif on p53 [29]. Our initial hypothesis was that SPRR2A contacts the CH3 domain of p300 and thereby precludes contact of p300 with other co-factors, like PCAF, thus preventing p300 acetylation. However, immunoprecipitation studies failed to reveal direct p300-SPRR2A binding. This led us to determine whether other molecules might mediate the p300 and p53 deacetylation.

Histone deacetylases do not act independently, but are recruited to complexes that regulate their deacetylase activity [30]. Gene array data showed that among the histone deacetylase superfamily, histone deacetylase 1 (HDAC1) was significantly upregulated in SPRR2A over expressing cells (data not shown). HDAC1 was an attractive candidate molecule for SPRR2A induced p53 deacetylation for the following reasons: 1) HDAC1 affects p53 acetylation through interactions with both p300 [31] and other cofactors such as MDM2 and mSin3a [7,32]; 2) HDAC1 acts as an antagonist of p53 in the regulation of p21 transcription [33]; 3) HDAC1 is known to complex with factors that mediate p53 ubiquitination [7], targeting p53 for proteosomal degradation and reducing total cellular p53 (Figure 1D) and; 4) HDAC1 is required for TGF-β1 induced EMT in hepatocytes [34] and SPRR2A overexpression induces EMT in cholangiocarcinoma cell lines [15].

**Figure 3:**
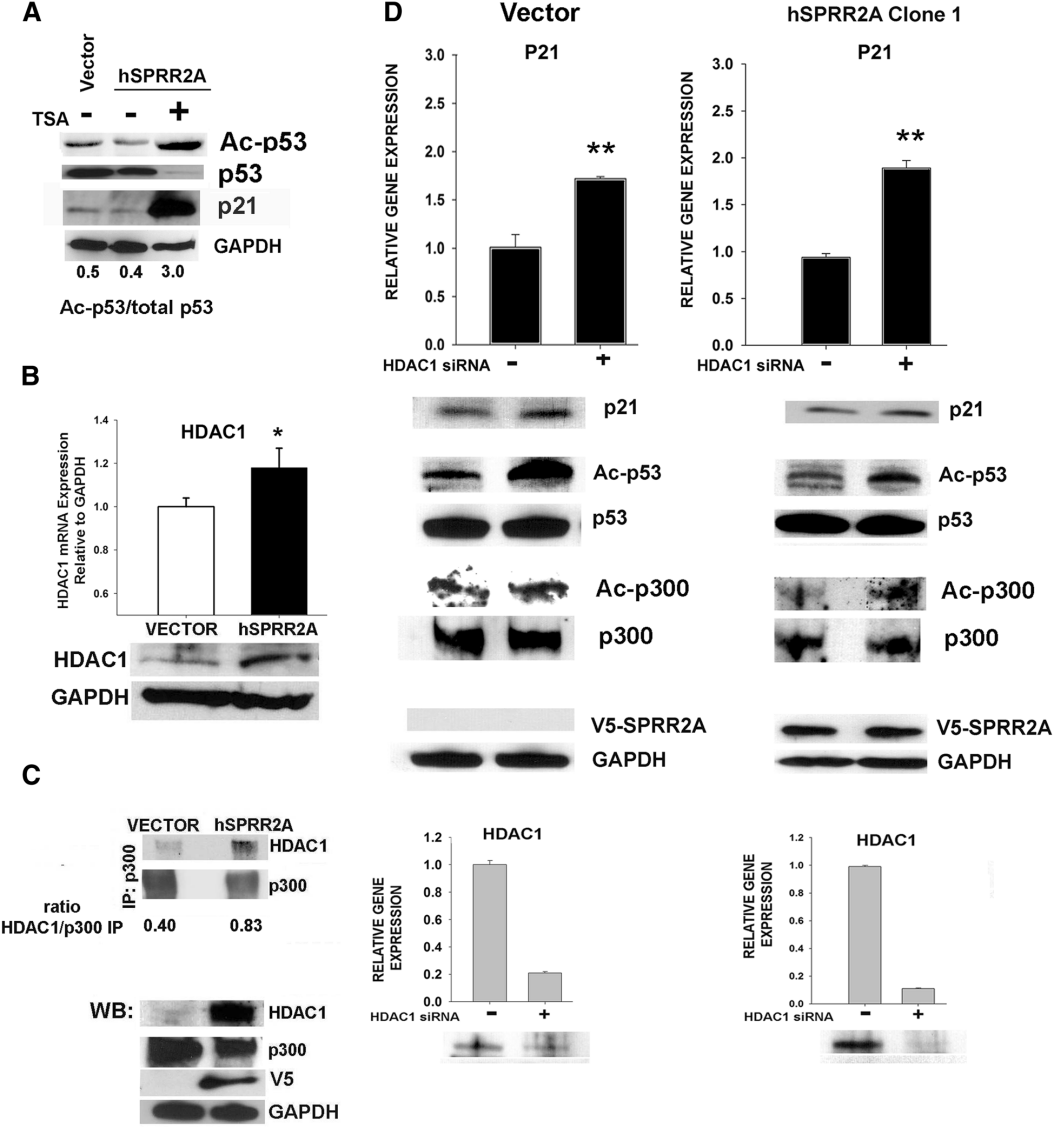
**HDAC1 modulates SPRR2A induced changes in p53 and p300 acetylation.** (**A**) Exposure to the deacetylase inhibitor trichostatin A (TSA) causes acetylation of most all of the p53 in SPRR2A cells, indicating a role for Class I and II deacetylases in the cellular affects of SPRR2A expression. Concomitant with this TSA increase in p53 acetylation is an increase in p21 expression. (**B**) HDAC1 mRNA and protein expression are increased in SPRR2A cells. (**C**) HDAC1 co-immunoprecipitates with p300 and there is more HDAC1/p300 binding in SPRR2A cells. (**D**) Knockdown of HDAC1 with siRNA: increases p21 protein and mRNA expression; restores acetylated p53 in SPRR2A cells to levels seen in the untreated vector control cells; and increases acetylated p300. Successful HDAC1 knock down was verified by both real-time PCR and western blotting. Real time PCR analysis: comparative 2-**ΔΔ**CT method (GAPDH internal control).

To determine whether histone deacetylases mediate a reduction of Ac-K382-p53 during SPRR2A over-expression, we used the deacetylase inhibitor trichostatin A (TSA), which globally interferes with Class I and II deacetylase activity. TSA is a powerful inhibitor of deacetylase activity and treatment of SPRR2A cells with TSA resulted in most all of the cellular p53 remaining in the acetylated form (Figure 3A). This indicates that SPRR2A-induced deacetylation of p53 can be reversed by class I/II deacetylase inhibition and that it is not controlled by a TSA-resistant NAD dependent histone deacetylase such as SIRT1 (class III) [35]. 

We next verified gene array data for HDAC1 by real time PCR and western blotting (Figure 3B). Over expression of HDAC1 interfered with p53 activation by binding to the CH3 domain of p300 and competitively inhibiting p53-p300 interactions [31]. Since SPRR2A-mediated p53 deacetylation and reduction of p21 expression required a functional p300 CH3 domain (Figure 2), we next determined whether HDAC1 binds to p300 in our cells. As shown in Figure 3C, endogenous HDAC1 co-immunoprecipitates with p300. Because SPRR2A cells over express HDAC1, there is more p300-HDAC1 interaction, competitively inhibiting p53-p300 binding.

We next inhibited HDAC1 expression using specific siRNA to determine whether HDAC1 was the specific deacetylase involved. Western blots show that reducing HDAC1 in SPRR2A cells restores acetylated K382-p53 levels (Figure 3D). Additionally, knockdown of HDAC1 recovered some p300 acetylation in SPRR2A cells. This agrees with a previous report that showed the association of deacetylases with p300 regulates its own acetylation status [31]. Finally, we show that HDAC1 siRNA not only increases Ac-K382-p53, but it increases p21 mRNA and protein expression (Figure 3D), implicating this molecule in the SPRR2A induced deacetylation of p53. Additionally, immunoprecipitation experiments determined that there were no direct HDAC1/SPRR2A protein interactions (data not shown).

## Conclusion

**Figure 4:**
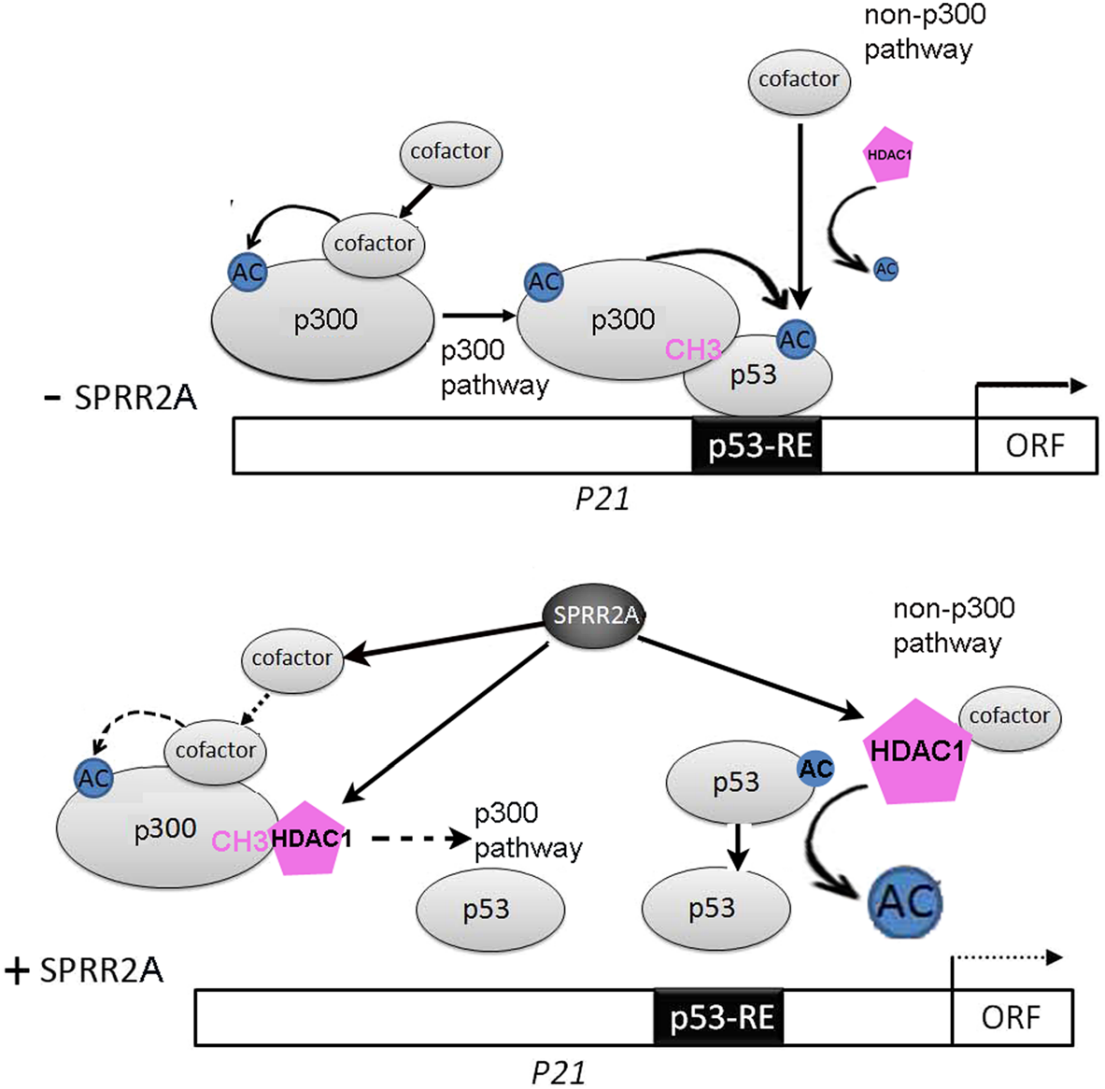
**A hypothetical model of how SPRR2A changes p53 acetylation and target gene transcription.** Acetylated p53 has a greater binding affinity to specific RE in the DNA, promoting target gene transcription. Upper diagram: Vector cells have lower HDAC1 deacetylase activity, allowing acetylated p300 and other modulatory proteins to enhance K-382-p53 acetylation. Lower diagram: Enhanced HDAC1 expression in SPRR2A cells directly deacetylates p53 and competitively inhibits acetylation through p300/p53 interactions. In addition, SPRR2A expression reduces p300 acetylation, most likely through its effects on other proteins involved with p300 auto-acetylation.

Our algorithm for reduced p53 acetylation and target gene transcription during SPRR2A over expression is outlined in Figure 4. SPRR2A induction of HDAC1, in combination with other cofactors, deacetylates Ac-K382-p53 and targets the protein for ubiquitination and subsequent degradation (non-p300 pathway). HDAC1 also competes with p53 for binding to acetyltransferase p300, reducing both p53 and p300 acetylation (p300 pathway). Although SPRR2A does not bind directly to p300, it might interfere with other cofactors involved with p300 autoacetylation. All molecular mechanisms for reduced p300 acetylation with SPRR2A over expression are not known, but cannot be solely explained by increasing HDAC1; further studies are needed.

Finally, p53-DNA binding is a critical event regulating gene expression during cellular stress, some of which might be disadvantageous during wound repair responses in barrier epithelia. For example, p53 transcriptional activation can trigger cell-cycle arrest, apoptosis, senescence, DNA repair, alter metabolism [36] and inhibit EMT [11]. SPRR2A, in contrast, functions as a suppressor of p53-dependent transcriptional activity by reducing the levels of acetylated p53. This deacetylation of p53 combined with the inherent antioxidant qualities of SPRR [18] protects SPRR2A expressing epithelial cells from damage and allows them to transiently acquire the mesenchymal characteristics needed for the restitution phase of wound repair.

## Materials and methods

### Cultured cells, and SPRR2A stable transfectants

The human intrahepatic cholangiocarcinoma cell line HuCCT-1 was maintained as reported [37]. Methods to obtain stable transfectants with a SPRR2A expressing vector were previously published [15].

### Plasmids

We used the C-terminal His-V5-tagged human SPRR2A expression vector previously described [15]. A human Halo-tagged p300 vector was purchased from Promega (Madison, WI). Other plasmids, including luciferase reporter plasmids, were purchased from Addgene (Cambridge, MA): luc-p21-promoter constructs; luc-p53-wt; luc-p53-mut; Ha-p300; and Ha-p300 CH3 deletion.

### Florescence imaging

The SPRR2A sequence was cloned into a DsRed mammalian expression vector (Addgene) and transfected into HuCCT-1 cells grown on glass coverslips. 48 hours after transfection, the coverslips were fixed for 1 hour in 1% paraformaldehyde. Nuclear staining was done with Hoechst dye. DsRed-SPRR2A and Hoechst florescence was captured using an AxioImager M1 microscope (Carl Zeiss, Gottingen, Germany) with a 40X objective lens, NA = 0.95.

### Biotinylated oligonucleotide precipitation assays

The probes for DNA pull-down assays are shown in Figure 2A. The assays were carried out as described [38]. Briefly, twenty four hours after transfection, cells were lysed with HKMG buffer (10 mM HEPES, pH 7.9; 100 mM KCl; 5 mM MgCl2; 10% glycerol; 1 mM DTT; and 0.5% of NP-40) containing protease and phosphatase inhibitors. Extracted proteins were pre-cleared (1 hr) with ImmunoPure streptavidin-agarose beads (Pierce, Rockford, IL). Pre-cleared lysates were then incubated 12 hours with 1 μg of the 5’-biotinylated double-stranded oligonucleotides and 10 μg of competitor DNA (poly(dI-dC) poly(dI-dC)) to eliminate non-specific protein/DNA interactions. Oligo-specific bound proteins were collected with streptavidin-agarose beads, separated by SDS-PAGE, and protein identification done by Western blotting.

### Transfections and luciferase reporter assay

Transfections with DNA plasmids or empty vector were done with Lipofectamine 2000 (Invitrogen, Carlsbad, CA) using the manufacturer’s recommended protocol for adherent cells. p300 (s4696) and HDAC1(s73) knock down transfections were done with target specific or negative control (negative siRNA #1) Silencer® Select siRNA (Ambion, Austin, TX) using RNAiMAX (Invitrogen). Luciferase assays were carried out with a Promega assay kit system 24 hours post-transfection and measured on a luminometer.

### Western blotting

Cell lysates were obtained using TNE buffer (50 mM Tris, pH 8.0; 150 mM NaCl; 10% v/v NP40; 2 mM EDTA) containing protease inhibitors 48 hours after treatments. Cytosolic and nuclear proteins were separated using an NE-PER extraction kit (Thermo Scientific, Rockford, IL). Proteins were separated by SDS-PAGE and visualized using enhanced chemiluminescence reagents (Pierce, Rockford, IL). Antibodies (clone) used are the following: p53 (DO1), GAPDH (0411), p300 (N15), and Ha (F-7) HDAC1 (H-51) (Santa Cruz Biotechnology, Santa Cruz, CA); V5 (Invitrogen); Halo (Promega); PCAF (C14G9), acetylated lysine (9441), and Ac-K382-p53 (Lys 382) (Cell signaling, Danvers, MA). Western blots were measured using imageJ software ( http://rsbweb.nih.gov/ij/).

### Immunoprecipitation

Cell lysates were obtained 48 hours post-treatment using TNE buffer containing protease inhibitors. Immunoprecipitation was done with appropriate antibody and protein-A Dynabeads® (Invitrogen) (for endogenous proteins) or HaloLink (Promega) magnetic beads (for plasmid transfected cells). Proteins were visualized by Western blotting.

### Real-time PCR

Total RNA was extracted from cells 24 hours post-transfection with HDAC1 siRNA using Trizol® (Invitrogen) and following the manufacturer’s instructions. P21 and HDAC1 expression was quantified by TaqMan® real-time PCR using specific primers (Applied Biosystems, Foster City, CA). SPRR2A was done with SYBR Green using previously described primers [39]. Gene expression was normalized to GAPDH using the comparative 2-ΔΔCT method, with expression levels in the untreated control set to a value of 1.0.

### Statistics

All statistical analyses were performed using SigmaStat software. A *P* value of < 0.05 was considered statistically significant, and all tests were two-tailed. All interval values are expressed as mean ± SD. Group comparisons were analyzed with Kruskal-Wallis ANOVA or one way ANOVA.

## Abbreviations

CH: Cysteine and histidine-rich region; HDAC1: Histone deacetylase 1; EMT: Epithelial-mesenchymal transition; SPRR2A: Small proline rich protein 2a, RE: response element.

## Competing interests

The authors declare that they have no competing interests.

## Author’s contributions

YM performed the majority of transfections and western blots in addition to the luciferase reporter and DNA pull down assays. SS conducted the immunoprecipitation, endogenous protein and HDAC1 experiments. KI performed the cellular staining. JL and NC participated in the study design and drafting of the manuscript. TT and AD were instrumental in the conceptual design of the study, evaluation of experiments and drafting of the manuscript. All authors read and approved the final manuscript.

## Supplementary Material

Additional file 1 **Figure S1.** SPRR2A deacetylates p53 in HepG2 cells, which express only wild type p53. (A) Real time PCR shows HepG2 cells do not normally express SPRR2A, but are successfully transfected with the SPRR2A plasmid. The (+) control was a SPRR2A stably transfected clone (cell line: SG231). (B) Western blot showing that transfection with SPRR2A in HepG2 cells reduces acetylation of K-382-p53. Endogenous p53 in HepG2 cells is wild type. (C) Real time PCR showing that SPRR2A transfection also reduces p21 mRNA expression. Real time PCR analysis: comparative 2-**ΔΔ**CT method (GAPDH internal control); ** p<0.01; ***p< 0.001. Click here for file
