# Supplementary figures and images for: SPRR2A enhances p53 deacetylation through HDAC1 and down regulates p21 promoter activity
Source: BMC Mol Biol. 2012 Jun 25;13:20. doi: 10.1186/1471-2199-13-20 (PMC3495018; doi:10.1186/1471-2199-13-20)

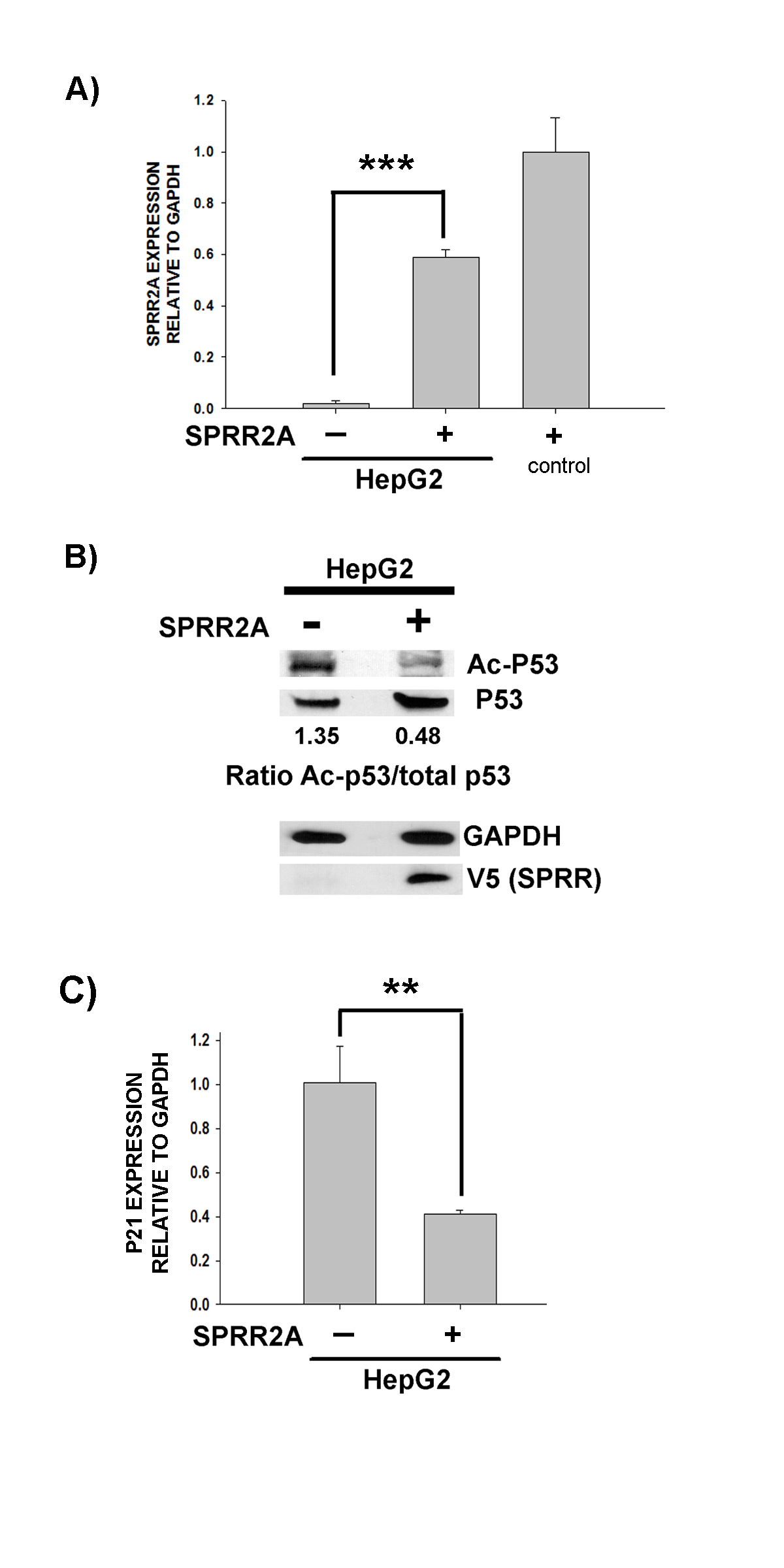

Supplement: Additional file 1 — Figure S1. SPRR2A deacetylates p53 in HepG2 cells, which express only wild type p53. (A) Real time PCR shows HepG2 cells do not normally express SPRR2A, but are successfully transfected with the SPRR2A plasmid. The (+) control was a SPRR2A stably transfected clone (cell line: SG231). (B) Western blot showing that transfection with SPRR2A in HepG2 cells reduces acetylation of K-382-p53. Endogenous p53 in HepG2 cells is wild type. (C) Real time PCR showing that SPRR2A transfection also reduces p21 mRNA expression. Real time PCR analysis: comparative 2-ΔΔCT method (GAPDH internal control); ** p<0.01; ***p< 0.001. [file 1471-2199-13-20-S1.tiff]
